# New onset neuromyelitis optica in a young Nigerian woman with possible antiphospholipid syndrome: a case report

**DOI:** 10.1186/1752-1947-2-348

**Published:** 2008-11-17

**Authors:** Morenikeji A Komolafe, Edward O Komolafe, Taofiki A Sunmonu, SO Olateju, CM Asaleye, Olufemi A Adesina, SA Badmus

**Affiliations:** 1 Department of Medicine, Obafemi Awolowo University Teaching Hospitals Complex, Ile-Ife, Nigeria; 2 Department of Surgery, Obafemi Awolowo University Teaching Hospitals Complex, Ile-Ife, Nigeria; 3 Department of Radiology, Obafemi Awolowo University Teaching Hospitals Complex, Ile-Ife, Nigeria

## Abstract

**Introduction:**

Devic's neuromyelitis optica is an inflammatory demyelinating disease that targets the optic nerves and spinal cord. It has a worldwide distribution and distinctive features that distinguish it from multiple sclerosis. There has been no previous report of neuromyelitis optica from our practice environment, and we are not aware of any case associated with antiphospholipid syndrome in an African person.

**Case presentation:**

We report the case of a 28-year-old Nigerian woman who presented with neck pain, paroxysmal tonic spasms, a positive Lhermitte's sign and spastic quadriplegia. She later developed bilateral optic neuritis and had clinical and biochemical features of antiphospholipid syndrome. Her initial magnetic resonance imaging showed a central linear hyperintense focus in the intramedullary portion of C2 to C4. Repeat magnetic resonance imaging after treatment revealed resolution of the signal intensity noticed earlier.

**Conclusion:**

Neuromyelitis optica should be considered in the differential diagnoses of acute myelopathy in Africans. We also highlight the unusual association with antiphospholipid syndrome. Physicians should screen such patients for autoimmune disorders.

## Introduction

Neuromyelitis optica (NMO) and the neuromyelitis spectrum disorders are inflammatory demyelinating disorders that affect the central nervous system (CNS) and specifically target the optic nerves and the spinal cord. The syndrome is characterized by a rapid or sub-acute severe bilateral visual loss accompanied by transverse myelitis and paraplegia. It tends to affect adults at an older median age compared with typical multiple sclerosis. Previously, NMO was regarded as a variant of multiple sclerosis, however, important distinguishing features include the absence of brain involvement on magnetic resonance imaging (MRI), the presence of extensive signal changes affecting more than three segments of the spinal cord and the presence in the serum of a specific autoantibody, Neuromyelitis Optica Immunoglobulin (NMO-IgG) [1-3]. The NMO-IgG is produced by the peripheral B cells and binds to Aquaporin 4, a CNS predominant water channel protein expressed on the astrocytic foot processes. These activate complement and initiate the autoimmune process and necrosis that is seen in the disease [4]. This new finding has important implications for treatment as rituximab, a B-cell specific monoclonal antibody may be effective in patients not responding to other treatments [5,6].

Neuromyelitis optica has a worldwide distribution and there are very few studies of the disease among Nigerian Africans. Osuntokun [7], in a review of hospital admissions at the University College Hospital Ibadan, Nigeria between 1957 and 1967 reported 95 cases of NMO with an estimated prevalence of 43 per 100,000 hospital cases.

The etiological factors that have been described include viral infections, tuberculosis and autoimmune disorders such as Sjogren's syndrome, systemic lupus erythematosus (SLE) and anti-phospholipid syndrome [8-10]. NMO occurring following the administration of vaccines prepared from whole, killed or live attenuated vaccines such as the pertussis, influenza and tetanus immunizations had been reported by Tezzon *et al*. [10].

The presentation of NMO could be monophasic or relapsing. The autoimmune disorders present with the relapsing type while that following immunization tends to be monophasic [11]. Previous workers have also noted that NMO could predate the manifestation of autoimmune disorders in some patients [11].

We are not aware of any previous report of NMO from our practice environment and in this brief presentation, we report a young Nigerian woman presenting with Devic's NMO and possible antiphospholipid syndrome.

## Case presentation

A 28-year-old Nigerian woman of Igbo ethnicity presented with a 6-week history of neck pain associated with paroxysmal tonic spasms of the left upper and lower limbs. She had no sphincter dysfunction or constipation. There was a positive Lhermitte's sign with neck flexion and severe burning sensation of the right lower limb. There was no prior neck trauma, cough, night sweats or weight loss and she did not complain of visual blurring. She was not hypertensive or diabetic but her father was hypertensive and her mother had diabetes. She was para 1 + 2 (1 alive) and had recurrent pregnancy losses twice in the mid-trimester period. She delivered a live male neonate 4 months before presentation and had a tetanus toxoid injection 3 days before presentation in addition to the three doses she had during antenatal care. She had a previous history of anterior neck swelling a month after delivery.

General physical examination showed a young woman with frequent paroxysmal tonic flexor spasms lasting 2 minutes each and involving the left upper and lower limbs. Higher mental function was normal. The Lhermitte's sign was elicited by forward neck flexion. The pupils were 3 mm in size and she had a relative afferent pupillary defect in the right eye. Initial fundoscopy was normal. She had a spastic quadriparesis with a power of grade 4 [Medical Research Council (MRC) grading] and bilateral extensor plantar response and absent abdominal reflexes. Light touch, vibration and joint position sensations were impaired up to the C7/C8 dermatome. Romberg's sign was present. Her cerebellar system was normal and there was no spinal tenderness. Her pulse rate was 80 beats/minute and regular. Her blood pressure was 120/80 mmHg with normal heart sounds. Chest and abdominal examinations were normal.

**Figure 1:**
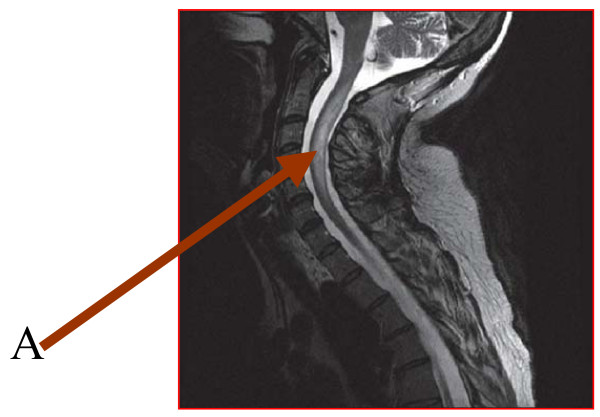
Cervical spine magnetic resonance imaging showing the hyperintense focus (Arrow A).

The cranial MRI showed normal T1 and T2 weighted images. The initial cervical T2 weighted MRI showed patchy ill-defined central linear hyperintense focus in the intramedullary portion of the spinal cord between C1 and C5 in keeping with transverse myelitis (Figure 1). Her erythrocyte sedimentation rate (ESR) on admission was 67 mm/hour and she had positive lupus erythematosus (LE) cells. Antinuclear and anti double-stranded antibodies were negative. Cardiolipin IgM was 15.0 MPL/ml (reference range 0.00–3.5 MPL/ml) while Cardiolipin IgG was within normal limits at 2.8 mGPL/ml (reference range 0.00–15.0 GPL/ml). Her prothrombin time (PT) was prolonged -PT 18.4s, control 12.9s, prothrombin time ratio (PTR) 1.4, international normalized ratio (INR) 1.6.

Her thyroid, liver function test, blood glucose and electrolytes were normal. The results were: serum calcium 2.4 mmol/liter, potassium 3.9 mmol/liter, sodium 135 mmol/liter, bicarbonate 26 mml/liter, chloride 98 mmol/liter and urea 4.5 mmol/liter. She was retroviral negative.

Her cerebrospinal fluid (CSF) test was normal: CSF protein 21 mg/dl, CSF glucose 2.8 mmol/liter, microscopy <5 WBC/mm^3^; CSF Immunoglobulin and serum NMO IgG assays could not be done.

An initial assessment of transverse myelitis was made and she was placed on intravenous methylprednisolone 1 g daily for 5 days, thereafter oral prednisolone 70 mg daily which was gradually tailed off. Although her serum potassium level fell to 2.9 mmol/liter, she did not receive additional potassium supplement with the therapy. She was however advised to take foods high in potassium. Her drug therapy included diazepam 15 mg 6 hourly and Baclofen 10 mg at night given for relief of the spasms. Gabapentin 400 mg at night and carbamazepine 400 mg thrice daily were also administered for the neuropathic pain. She also had regular physiotherapy.

The patient gradually improved with gradual resolution of the muscle spasms, weakness and rigidity. Muscle power increased to 5 globally and the repeat ESR reduced to 17 mm/hour.

Ophthalmic examination done 4 weeks after admission revealed impaired visual acuity which was worse in the right eye (right eye – counting figure, left eye – 6/9). The corneal sensitivity was intact with a relative afferent pupillary defect. There was bilateral temporal pallor with slight blurring of the optic disc margins nasally. There were also nerve fiber defects along the superotemporal vascular arcade with moderate perivascular sheathing.

**Figure 2:**
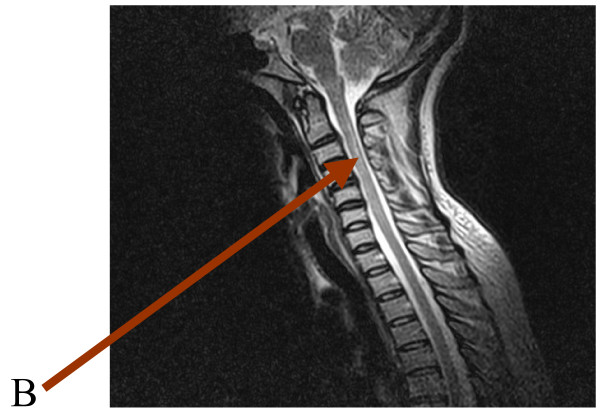
Cervical spine magnetic resonance imaging post-treatment with resolution of the previously noticed focus (Arrow B).

A repeat MRI was done a month after the initial one and showed resolution of the earlier noticed signal intensity at the spinomedullary junction (Figure 2). A final diagnosis of Devic's NMO syndrome was made and she was discharged after 5 weeks to be followed up in the outpatient clinic. She has been seen many times at the clinic and there has been sustained clinical and neurological improvement. However, she did not receive additional immunosuppressive therapy after completing the intravenous methylprednisolone, neither was there a repeat confirmatory test done for her antiphospholipid status. She was followed up in the clinic for 8 months after her discharge.

## Discussion

Devic's NMO is characterized by a unilateral or bilateral optic neuritis and transverse myelitis, with a variable interval between the two events. It is usual for the optic neuritis to precede the myelitis but, in this patient, it is interesting to note the myelitis preceding ophthalmic features of optic neuritis. The association of the presentations with features of antiphospholipid syndrome (APS) is also an unusual presentation. The patient had previous abortions, positive LE cells and a prolonged PT with the presence of IgM Cardiolipin antibody. This suggests the possible presence of a secondary type of antiphospholipid syndrome; however, she did not fulfill the research criteria for APS.

Antiphospholipid syndrome is also an autoimmune disorder with autoantibodies affecting a wide variety of organs including the spinal cord. She also had evidence of autoimmune thyroiditis with a transient neck swelling. It is also unusual that the CSF parameters were normal, although facilities for immunoglobulin analysis were not available. In NMO, a positive antinuclear antibody status may be present without evidence for systemic connective tissue disease. Similarly too, a positive APS antibody status is found without clinical features of the disease. Some speculate that this represents positive autoantibodies that occur as a result of the general autoimmune tendency. There might also be no evidence of autoantibodies at the onset of illness, but these may occur after several years with classical features of SLE and APS. An example is a 37-year-old woman reported by Jacobi *et al. *[11] with recurrent episodes of transverse myelitis and optic neuritis which were followed years later by clinical and laboratory findings diagnostic for SLE.

The role of the tetanus toxoid received is also an important point to note in this patient. Active or passive immunization with vaccines or sera can cause lesions in the central and peripheral nervous systems. Tezzon *et al. *[10] also reported a case of transverse myelitis with radicular component which occurred acutely following administration of tetanus toxoid with the patient having a partially favorable outcome. Hence tetanus toxoid immunization might also play a role in the pathogenesis of NMO in this patient. This case further underscores the importance of readily available neuroimaging in arriving at a definite diagnosis and choosing appropriate treatment. This patient was earlier managed for cervical spondylosis from the referral centers because of the moderate degenerative changes seen on plain X-rays of the cervical spine. Hence a high index of suspicion and early and appropriate neuroimaging will further enhance appropriate diagnosis and treatment as well as improve the outcome.

## Conclusion

In conclusion, neuromyelitis optica (NMO) may be associated with features of autoimmune disorders such as antiphospholipid syndrome and systemic lupus erythematosus. It is suggested that all patients with NMO be screened for autoimmune disorders and aggressive treatment should be commenced after a thorough laboratory work-up.

## Abbreviations

CSF: cerebrospinal fluid; INR: international normalized ratio; NMO: neuromyelitis optica; MRI: magnetic resonance imaging; NMO Ig: neuromyelitis optica immunoglobulin; CNS: central nervous system; APS: antiphospholipid syndrome; SLE: systemic lupus erythematosus; C7/C8: seventh and eighth cervical spine; C2: second cervical spine; C4: fourth cervical spine; T1: first thoracic spine; T2: second thoracic spine; ESR: erythrocyte sedimentation rate; CSF: cerebrospinal fluid; WBC: white blood cells; PT: prothrombin time; PTR: prothrombin time ratio; PTTK: partial thromboplastin time of kaolin; MRC: Medical Research Council; LE: lupus erythematous; MPL/ml: unit of affinity purified IgM per milliliter; GPL/ml: unit of affinity purified IgG per milliliter

## Consent

Written informed consent was obtained from the patient for publication of this case report and any accompanying images. A copy of the written consent is available for review by the Editor-in-Chief of this journal.

## Competing interests

The authors declare that they have no competing interests and confirm that all authors have seen and agreed the content of the manuscript. This work has not been submitted or published elsewhere.

## Authors' contributions

All of the authors were involved in the management of the patient. OO the ophthalmologist managed the optic neuritis. AC the radiologist interpreted the MRI. KM and KE both participated in the preparation of the manuscript. All authors read and approved the final manuscript.
